# Implementation fidelity of hospital based directly observed therapy for tuberculosis treatment in Bhutan: mixed-method study

**DOI:** 10.1186/s12889-020-08666-w

**Published:** 2020-04-19

**Authors:** Kunzang Dorji, Trisasi Lestari, Sonam Jamtsho, Yodi Mahendradhata

**Affiliations:** 1 International Master program in Public Health, Faculty of Medicine, Public Health and Nursing, Universitas Gadjah Mada, Yogyakarta, Indonesia; 2 Present Address: Royal Centre for Disease Control, Department of Public Health, Ministry of Health, Thimphu, Bhutan; 3 Center for Tropical Medicine, Faculty of Medicine, Public Health and Nursing, Universitas Gadjah Mada, Yogyakarta, Indonesia; 4 Phuentsholing General Hospital, Chukha, Bhutan; 5 Department of Health Policy and Management, Faculty of Medicine, Public Health and Nursing, Universitas Gadjah Mada, Yogyakarta, Indonesia

**Keywords:** Direct observed treatment, Implementation fidelity, Tuberculosis Bhutan

## Abstract

**Background:**

Direct observed treatment (DOT) has been implemented in Bhutan since 1997 and currently, it is offered in various model of delivery including a combination of hospital based, home based DOT and ambulatory DOT. Overall, treatment success rate for tuberculosis cases is higher than the global target; however, it is still need to be improved. Evaluation to the implementation fidelity of DOT is important to identify potential rooms for improvement. This study aimed to assess two major components of the program’s implementation fidelity: to assess patient’s adherence to DOT and explore factors for adherence; to assess provider’s compliance with DOT guideline and explore factors for compliance.

**Methods:**

This research used a sequential explanatory mixed method. The conceptual framework of implementation fidelity was adopted to guide this study design. The cross-sectional study of TB patients was enrolled in two hospitals with highest TB load, between September to November 2017 in Bhutan. Interviewer assisted survey was conducted with 139 TB patients who visited the hospital in continuation phase. In-depth interview was then conducted with nine TB patients and four health staffs to explore the barriers and enablers of DOT.

**Results:**

Total of 61.9% (86/139) of patients has received DOT at intensive phase. Proportion was higher among MDR-TB cases (100%), and smear sputum positive TB cases (84.7%). In the continuation phase, 5.8% of patients took medicine at hospital, 48.9% at home and the rest 45.3% no longer practiced DOT. More than 90% of patient received correct dosage and standard regimen of anti-TB drugs according to the guideline. The key factors affecting poor adherence to DOT as perceived by patients were; lack of willingness to visit the clinic on daily basis due to long distance, financial implications and family support. However, patient’s satisfaction to the quality of TB treatment service delivery was high (98.6%). Providing incentives to the patient was most agreed enabler felt by both health workers and patients.

**Conclusion:**

In the selected hospital sites, the patient’s adherence to DOT and provider’s compliance with DOT guideline is partially implemented; the coverage and the duration of DOT is very low, therefore, need to revise and improve DOT model and structure.

## Background

Tuberculosis (TB) remains a major public health problem in Bhutan, especially with a rise of multi-drug-resistant TB cases (MDR-TB). It is estimated that 27% of the total population in Bhutan are infected with *Mycobacterium tuberculosis* and this leads to high incidence of TB (178 per 100,000 population) [1]. In 2016, there was a total of 1143 TB cases reported, of which 79.4% is in productive age between 15 and 59 years. Compare to Bhutan’s TB data in the last 5 years, number of TB cases was slightly increasing [2]. In 2016, treatment success rate was 91.6%, failure rate was 4.6%, mortality rate was 2.4%, and defaulted rate was 0.3% [3]. Despite high treatment success rate, MDR-TB cases is rising dramatically from just six in 2005 to 55 in 2016. Proportion of MDR-TB cases among new TB cases was higher (11%) compare to the neighboring countries, i.e. China (7.1%), India (2.8%) and Nepal (2.2%) [4]. High proportion of MDR-TB cases among new cases indicates that MDR-TB is being transmitted as primary infection in the general population. The proportion of MDR-TB was even higher among previously treated cases (18%). All MDR-TB cases were tested for resistance to second-line drugs and until this study was conducted there were no extensively drug-resistant tuberculosis (XDR-TB) cases identified [5].

The National Tuberculosis Control Program (NTCP) was launched in 1997 and fully integrated into the health system. The health system consists of a three-tiered health system, which comprises one National Referral Hospital, which is the Jigme Dorji Wangchuck hospital, two Regional Referral Hospital located at Mongar in the east and Gelephu in the central region, and 29 districts hospitals. At the community level, health care services are provided through 201 Basic Health Units (BHU). TB diagnostic and treatment services are provided through all type of hospitals and five selected BHU. Other BHUs are involved in screening and referral of presumptive TB cases; follow up, default and contact tracing and provision of Directly Observed Treatment (DOT). Initiation of TB treatment are made in a hospital and treatment is observed directly by hospitalization of all TB cases during intensive phase. In the continuation phase, the patient is discharged and followed up by a DOT provider from the BHUs. TB patient who lives close to a hospital or a BHU can be treated as out-patient at health facilities [1]. This model has been implemented since 1997. However, a previous study of MDR-TB in Bhutan revealed that 74% of MDR-TB cases have previous history of anti-TB treatment; and 79% of these cases did not comply with DOT [6]. Hence, there is a need to improve DOT implementation fidelity, by understanding the barriers and facilitators for implementing DOT according to the guideline [7, 8]. Therefore, this study aims to evaluate implementation fidelity of DOT model in Bhutan using the conceptual framework for implementation fidelity.

## Method

### Study setting

This study was conducted at Jigme Dorji Wangchuk National Referral Hospital (JDWNRH) in Thimphu district, and Phuntsholing General Hospital (PGH) in Chukha district, Bhutan. These two study sites were selected because they are located in a high burden of TB district and treated more TB cases compared to other hospitals in the country [1].

### The intervention and implementation strategy

Bhutan’s DOT model combines TB intensive care and observation in hospital at least for the first 2 weeks of treatment for all smear sputum positive TB patients, and clinic based or home based direct observation during the continuation phase. As soon as diagnosis of TB is confirmed, treatment will be started at a health facility for 6–8 months depending upon the TB category [1]. Provide/ arrange DOT to TB patients for the entire duration of TB treatment [1]. A smear positive pulmonary TB cases will be encouraged to be admitted for at least 2 weeks in the isolation room. Longer hospitalization will be needed for severely ill patient or patient with serious co-morbidity. Most patients will receive ambulatory DOT on daily basis at the nearest health facility from patient’s workplace or home. The DOT provider will be a facility based healthcare worker. Patient who lives far from hospital or BHU will be offered home based DOT on daily basis provided by a healthcare worker or a trained and supervised village health worker who is selected by the medical officer and TB coordinator in the facility, following consultation with the patient and patient’s family along with the village health worker. Patient’s identity, address, phone number and DOT activities are recorded on the patient’s treatment card and DOT provider will have the filled in copy of patient’s treatment card [1].

### Study design

This study has adapted the implementation research type with IR outcome of measuring fidelity. Implementation research study is form of research that addresses implementation bottlenecks, identifies optimal approaches for a particular setting, and promotes the uptake of research findings [9]. Implementation research aims to cover a wide set of research questions, implementation outcome variables, factors affecting implementation, and implementation strategies [10]. While the Implementation itself means “the process of putting to use or integrating evidence-based interventions within a specific setting” [11]; at least eight conceptually distinct implementation outcomes- acceptability, adoption, appropriateness, feasibility, fidelity, implementation cost, penetration, and sustainability [11].

We have used mixed-method explanatory sequential design that adapts Conceptual Framework of Carroll for assessment of implementation fidelity. This study used a mixed method explanatory sequential design, which involved quantitative study followed by qualitative study. We have chosen mixed method explanatory sequential design because the qualitative is needed to explain quantitative findings. The study starts with a cross-sectional survey to assess patient’s characteristics, patient’s adherence to treatment, and providers’ compliance towards implementation of DOT.

### Conceptual framework

**Fig. 1:**
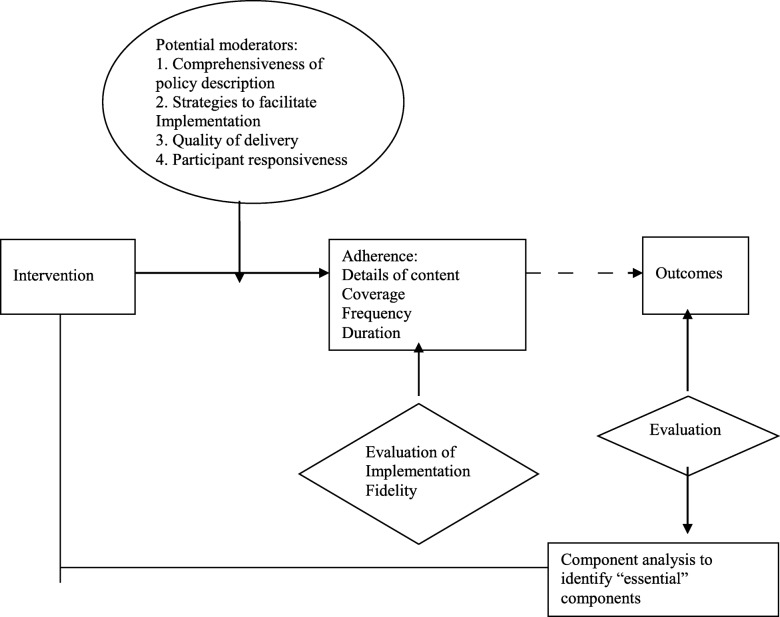
Conceptual framework for implementation fidelity

This study was guided by the conceptual model for implementation fidelity from Carroll et al. [8]. All components of adherence to DOT guideline (coverage, content, frequency, and duration), and the moderating factors, which comprise intervention complexity, facilitation strategy and quality of delivery were measured (Fig. 1).

Coverage is defined as characteristics and proportion of TB patient actually received DOT according to the guideline. In other words it means proportion (%) of patients who received DOT for at least part of the whole treatment duration. Content is defined as compliance to the national guideline of TB management, i.e. provide correct dosage and correct regimen and ensure continuity of treatment. The DOT guideline also recommends that providers help patients comply with the treatment regimen as much as possible through education of patients and their families, providing nutritional support and addressing social determinants of TB such as stigma, social isolation, interruption of studies, loss of employment and other negative consequences of TB.Frequency is defined as interval between DOT visit, and duration is defined as the length of time of which DOT was provided to the patient.

Three moderators that may influence the degree of fidelity were defined as follow: 1. Intervention complexity was defined as existing barriers in program delivery; 2. Facilitation strategy was defined as any strategies to optimize implementation; 3. Quality of delivery was defined as quality impact score measured using the QUOTE TB light and patient’s satisfaction [12].

In the second phase of the study, we employed phenomenological study to describe in detail about the experience of TB patients and healthcare staff during program delivery and explain findings from the previous quantitative survey, as well as to explore barriers and enablers of implementation of DOT service, as perceived by both TB patients and service providers.

### Study participants and sample size

The quantitative study includes all TB patients at all ages, including MDR-TB patients, who were receiving treatment during continuation phase at the JDWNRH and PGH from September to November 2017. Total sampling was done for 3 months and we have enrolled a total of 139 TB patients, mostly adult age 15–49 years old. Number of child TB cases were very small (5 children) and parents were invited to participate in the study.

Participant for the qualitative study was selected purposively to represent all the categories of TB and health workers which include healthcare worker who have long experienced in serving TB patients; TB patients were (new cases, retreatment and MDR-TB) who already on treatment for more than 2 months, and TB program coordinator from the NTCP. Two healthcare workers from each hospital, three patients for each patient criteria and one TB coordinator from the NTCP were selected as respondents.

### Data collection and analysis

The questionnaires (which included both intensive and continuation phase) were distributed to TB patients and healthcare staffs to measure key components of adherence: implementation’s content, coverage, duration, dosage, and frequency; the quality of TB care services delivery, and satisfaction level towards TB service. The questionnaire was adapted and developed from the QUOTE TB Light tool [12],This is a questionnaire to measure quality of TB care based on the following quality dimensions: 1. Communication and information; 2. Professional competence; 3. Availability of TB DoT services; 4. Patient-Provider interaction and counseling; 5. Support; 6. Affordability; 7. TB-HIV; 8. Infrastructure and 9. Stigma. It was developed by TB CAP, in collaboration with KNCV at Uganda and Netherlands Institute for Health Research.

In quantitative phase, questionnaire survey was conducted by PI and two research assistants at TB clinic in both hospitals. Research assistants provide information about the study to potential respondent, obtained written consent, and assist the respondent in completing the questionnaire. Each questionnaire took about 20–25 min to complete. Returned questionnaire was then checked for completeness and double entered into an EpiData© (Version 3.1) database. We have cross-checked their treatment card and record book. We also verified the phase of the patient treatment by checking the TB register.

Analysis was performed using Stata© software (Version 13.1). Pearson Chi-Square test was used to see the statistical significance between the dependent and independent variables. Scores of quality of TB care service delivery was calculated using Microsoft Excel©. The heart of QUOTE TB Light is the quality impact (QI) score. QUOTE TB LIGHT is an acronym for “Quality of care as seen through the Eyes of the Patient”, the QUOTE TB LIGHT tool is ready to use since it was already tested quantitatively and validated through statistical analysis by TB CAP, in collaboration with KNCV at Uganda and Netherlands Institute for Health Research (NIVEL) [9]. These scores are calculated by combining the importance ranking based on health staff’s opinion and average performance scores according to patients’ survey. The higher the QI score, the more need for improvement. In general QI scores above 0.75 indicates that improvement is possible and may be necessary. A cutoff points was established to define the degree of implementation fidelity of DOT based on the percentage of maximum score achieved in relation to the expected total score for each sub-dimension: < 40% - Not implemented (Poor); 40 to < 75% - Partially implemented (moderate); and > 75% - Implemented (good).

In qualitative phase, a total of 14 semi-structured indepth interviews were conducted using local language (Dzongkha, Sharchop, and Nepali) and all interviews were led by the principal investigators. The interview questionnaires were constructed by PI and all co-authors based on NTCP TB guideline (see additional file 1 and additional file 2). Interviews with patients and heathworkers were conducted at hospital in seperate room away from TB treating room; and interviews with the NTCP representative was conducted at Ministry of Health. Informed consent were obtained from all respondents prior to the interviews. We asked open ended questions, eliciting each respondents experience in receiving or delivering DOT. This qualitative research has helped in quantitative method by identifying the barriers and facilitators in DOT delivery to assess four components of adherence and the moderating factors. Some of the questions are kept open-ended qualitative method so that patients can explain in detail. All interviews were digitally recorded and hand notes were made by the investigator and research assistant. All interviews were transcribed verbatim by research assistant and then translated into English. Qualitative data analysis was performed using OpenCode 4.03 software to generate codes, themes and major themes.

### Ethics consideration

Ethics clearances were obtained from the Ethics Committee of Faculty of Medicine, Universitas Gadjah Mada, Indonesia, and Research Ethics Board of Health (REBH) of Bhutan. Study permission was obtained from JDWNRH and PGH. Informed consent was obtained from all respondents prior to participation. To protect patient’s privacy, name was not included in questionnaire form and medical record number was decoded.

## Result

A total of 139 TB patients were participated in the quantitative study and 14 respondents, comprising of TB patients, healthcare workers, and NTCP coordinator were participated in the qualitative study. The core components of the conceptual model for implementation fidelity was measured and described below.

### Adherence

#### Coverage

Overall, DOT was provided to 87.1% of participants in various model. The proportion of patient receiving DOT was higher during the intensive phase than in the continuation phase (87.1% vs 54.7%). Hospital based DOT at intensive phase was provided to only 61.9% of participants. Proportion was higher among MDR-TB cases (100%), and smear sputum positive TB cases (84.7%). PGH, a district referral hospital, provided more hospital based DOT compare to JDWNRH, a national referral hospital (85.7% vs 58.9%). Only few cases received ambulatory DOT and almost one third of cases choose to have home based DOT since the beginning of treatment. In the continuation phase, very few patient continue taking medicine at the hospital under DOT (5.8%), and most patient took medicine at home and observed by family member (48.9%), the rest (45.3%) were no longer practiced DOT (Tables 1 and 2).

**Table 1:** Characteristics of study participants and DOT coverage

Variables	Total	Intensive Phase		Continuation Phase	
	n (%)	DOT	No DOT	DOT	No DOT
Gender					
Male	58 (41.7)	31 (53.4)	27 (46.6)	5 (8.6)	53 (91.4)
Female	81 (58.3)	55 (67.9)	26 (32.1)	3 (3.7)	78 (96.3)
Age					
< 14 year	5 (3.6)	2 (40)	3 (60)	0	5 (100)
15–49 year	123 (88.5)	81 (65.9)	42 (34.1)	8 (6.5)	115 (93.5)
> 50 year	11 (7.9)	3 (27.3)	8 (72.7)	0	11 (100)
Residence					
Urban	98 (70.5)	64 (65.3)	34 (34.7)	7 (7.1)	91 (92.9)
Urban slum	22 (15.8)	12 (54.5)	10 (45.5)	1 (4.5)	21 (95.5)
Rural	19 (13.8)	10 (52.6)	9 (47.4)	0	19 (100)
Highest Education Level					
> Diploma	38 (27.3)	20 (52.6)	18 (47.4)	4 (10.5)	34 (89.5)
Secondary	53 (38.1)	34 (64.2)	19 (35.8)	2(3.8)	51 (96.2)
Primary School	12 (8.6)	8 (66.7)	4 (33.3)	1(8.3)	11 (91.7)
No Education	36 (25.9)	24 (66.7)	12 (33.3)	1(2.8)	35 (97.2)
Occupation					
Civil servant	8 (5.8)	2 (25.0)	6 (75.0)	0	8 (100.0)
Private employee	35 (25.2)	28 (80.0)	7 (20.0)	4 (11.4)	31 (88.6)
Corporate employee	5 (3.6)	3 (60.0)	2 (40.0)	1 (20.0)	4 (80.0)
Armed Force	7 (5.0)	5 (71.4)	2 (28.6)	0	7 (100.0)
Student	28 (20.1)	15 (53.6)	13 (46.4)	2 (7.1)	26 (92.9)
Others	56 (40.3)	33 (58.9)	23 (41.1)	1 (1.8)	55 (98.2)
Type of TB					
SPPT	72 (51.8)	60 (83.3)	12 (16.7)	3 (4.2)	69 (95.8)
SNPT	15 (10.8)	3 (20.0)	12 (80.0)	1 (6.7)	14 (93.3)
EPT	39 (28.1)	11 (28.2)	28 (71.8)	0	39 (100.0)
MDR-TB	13 (9.3)	12 (92.3)	1 (7.7)	4 (30.8)	9 (69.2)
Treatment category					
New	125 (89.9)	74 (59.2)	51 (40.8)	5 (4.0)	120 (96.0)
Retreatment	14 (10.1)	12 (85.7)	2 (14.3)	3 (21.4)	11 (78.6)

*DOT* Directly observed treatment, *EPT* extra pulmonary tuberculosis, *MDR-TB* multi-drug-resistant tuberculosis, *SNPT* smear negative pulmonary tuberculosis, *SPPT* smear positive pulmonary tuberculosis

**Table 2:** Patient characteristics and type of DOT

		JDWNRH *N* = 111		PGH *N* = 28		Ambulatory		Home-based	
	Total	Int	Cont	Int	Cont	Int	Cont	Int	Cont
		N %	N %	N %	N %	N %	N %	N %	N %
All Cases	139	65 (58.6)	3 (2.7)	21 (75.0)	5 (17.9)	5 (3.6)	8 (5.8)	48 (34.5)	68 (48.9)
Type of TB									
SPPT	72	48 (85.7)	3 (5.4)	12 (75.0)	0	2 (2.8)	3 (37.5)	9 (12.5)	39 (57.4)
SNPT	15	2 (14.3)	0	1 (100.0)	1 (100.0)	0	1 (12.5)	11 (73.3)	6 (8.8)
EPT	39	9 (25.8)	0	2 (50.0)	0	3 (7.7)	0	28 (71.8)	18 (26.5)
MDR-TB	13	6 (100.0)	0	6 (85.7)	1 (14.9)	0	4 (50.0)	0	5 (7.4)
Age group									
0 to 14	5	0	0	0	0	0	0	2 (40.0)	4 (5.9)
15 to 49	123	63 (63.0)	3 (3.0)	20 (80.0)	5 (20.0)	5 (4.1)	8 (100.0)	40 (32.5)	60 (88.2)
≥ 50	11	2 (22.2)	0	1 (50.0)	0	0	0	6 (54.5)	4 (5.9)
Treatment category									
New	125	58 (56.9)	3 (2.9)	16 (69.6)	2 (8.7)	5 (4.0)	5 (62.5)	45 (36.0)	62 (91.2)
Retreatment	14	7 (77.8)	0	5 (100.0)	3 (60.0)	0	3 (37.5)	3 (21.4)	6 (8.8)
Gender									
Male	58	22 (48.9)	2 (4.4)	9 (69.2)	3 (23.1)	3 (5.2)	5 (62.5)	24 (41.4)	25 (36.8)
Female	81	43 (65.2)	1 (1.5)	12 (80.0)	2 (13.3)	2 (2.5)	3 (37.5)	24 (29.6)	43 (63.2)

Cont- continuation phase

EPT- extra pulmonary tuberculosis

Int- intensive phase

JDWNRH- Jigme Dorji Wangchuk National referral hospital

MDR-TB- multi-drug-resistant tuberculosis

PGH- Phuentsholing General Hospital

#### Content

More than 90% of patient received standard regimen of anti-TB according to the guideline. Treatment duration was modified to 9 to 12 months for 11 TB cases (7.9%) due to perceived risk of mono/poly resistance. All patients had received TB drugs on daily basis. The dosage was prescribed as per the NTCP guideline and in accordance with their weight range. i.e. two tablets of FDC for body weight less than 35 kg, three tablets for 35–54 kg and four tablets for more than 55 kg. Drug observer during intensive phase was healthcare worker (61.8%), family member (25.1%) and patient themself i.e. self administered (12.9%) (See Table 3).

**Table 3:** Compliance to the National TB management guideline

Variables	Total	JDWNRH	PGH
	n (%)	n (%)	n (%)
Standard treatment regimen			
New Cases- 6 month rifampicin (2HRZE+4HR)	105 (75.5)	85 (76.5)	20 (71.4)
Retreatment cases- 8 month rifampicin (3HRZE+5HRE)	10 (7.1)	9 (8.1)	1 (3.57)
Modified- 9 to 12 months	11 (7.9)	11 (9.9)	0
MDR Treatment- 18 to 20 months	13 (9.3)	6 (5.4)	7 (25)
Frequency			
Daily	139 (100)	111 (100)	28 (100)
Three times a week	0	0	0
Dosage as per body weight			
Correct dosage	(100)	(100)	(100)
Incorrect dosage	0	0	0
Directly observed treatment			
Yes	106 (76.2)	82 (73.8)	24 (85.7)
No	23 (16.5)	22 (19.8)	1 (3.5)
Sometimes	10 (7.1)	7 (6.3)	3 (10.7)
DOT intensive phase			
Health staff	86 (61.9)	65 (58.5)	21 (75.0)
Family member	35 (25.1)	30 (27.0)	5 (17.8)
None	18 (12.9)	16 (14.4)	2 (7.1)
DOT continuation phase			
Health staff	8 (5.7)	3 (2.7)	5 (17.8)
Family member	68 (48.9)	51 (45.9)	17 (60.7)
None	63 (45.3)	57 (51.3)	6 (21.4)

*DOT* Directly observed treatment, *JDWNRH* Jigme Dorji Wangchuk National referral hospital, *PGH* Phuentsholing General Hospital

#### Frequency

DOT in hospital was given in daily basis, Nurse visit TB patient in their bed every morning with the anti-TB drug and watch patient swallow the drug. Patient who choose to have ambulatory DOT, visited a hospital clinic (3.6%) in daily basis. Only 3.6% of patient who choose ambulatory DOT complied to the agreed rule to visit the clinic in daily basis.

*“Yes i take while they observe, they make us take medicine in front of them only”* (TB patient TH4). Another respondent *“Yes there is big difficulty in taking MDR medicine but there is no option, we have to take medicine in front of them only, never missed any drug as prescribed by doctor”* (TB patient TH5).

#### Duration

In average, TB patients received hospital based DOT for two weeks during initial intensive phase, ranging from one week to four months. The maximum duration of hospital-based DOT offered to SPPT was only for two weeks.

### Potential moderators

#### Intervention complexity

We found national health policy as the top comprehensive policy of the program, followed by NTCP guideline for both TB and MDR-TB case management as per the WHO TB treatment guideline. There was no separate standard operating procedure (SOP) for DOT maintained in the respective DOT centers during assessment.

*“Yes yes, we have. First slightly we followed WHO guideline, then NTCP guideline, SOP for each procedure like DOT and so on”* (Service provider1 TH).

Interviews with TB health staff and TB program officers revealed that they could not provide DOT service to all TB cases, due to unwillingness of the TB patients, thus the compliance towards DOT guideline was challenging for them. DOT was best practiced only when TB patients were hospitalized. Once they are discharged DOT was not monitored or followed. It is the responsibility of the patient to continue the treatment. Health provider does not know whether they strictly implement DOT or not.

*“… how much we try but the patient can't come daily for DOT purpose, only we can pursue the patient for DOT for two weeks only, after that they can't follow”* (Service provider1 TH).

*“… Since the inception DOT has been introduced in 1997 and the implementation of DOT in a real sense has not really picked up…”* (NTCP program officer1 TH).

We found the major barriers for poor adherence towards DOT as perceived by patients, service providers and NTCP program were; patients not willing to come to the clinic for DOT on daily basis due to long distance, financial crisis for traveling (cost for transportation and accomodation) and lack of support from family and friends. The health staff also feel that it is a burden for the patient to be admitted for a long period of time in a hospital. Other important factors were patient movement from place to place, and side effects of the drug.

Provider’s factors also plays role in the implementation of DOT. Hospital based DOT is not a compulsary, however, TB staff can encourage patient to undergo hospital based DOT for 2 weeks. Priority was given only to smear sputum positive TB patient, MDR-TB patient and severely ill patient. Interviews with health staff also revealed poor compliance to the selection guideline of DOT provider after patient discharged from the hospital. It is described by health providers as follows:

*“DOT is implemented in the hospital, but once the patient is discharged or upon conversion to sputum negative, it is the responsibility of the medical officer and TB staff in charged to consult the patient. They have to identify a DOT provider for the entire course of treatment, but when it comes to reality it is not 100% implemented”* (Program staff1).

“...*Before we had committee members now we directly hand over (the patient) to their relatives or to their friends with mobile numbers*” (Service provider1 TH).

#### Facilitation strategy

To improve adherence to DOT service, the NTCP had provided TB training for new TB staff, and refresher training to all relevant health workers, i.e. Nurse, Doctors and laboratory staff. The NTCP also sent TB incharge to outside-country training for 5 days. The NTCP also provided Nu. 100 (US$1.5) per month to all TB staff in 32 DOT centers as a compensation to follow up TB patients through call. All logistics, including TB drugs and reporting forms, were provided by the NTCP and distributed to all DOT centers.

*“We have also sent them for training to outside country for 5 days to keep same working spirit”* (NTCP program officer1).

Health providers felt that poor adherence to DOT could be aslo due to lack of incentives for the provider and the patients, because there is no compensation for wage loss and cost for transportation.

*“If they really want to improve DOT, then they have to give something like incentives to patients those who live far distance”* (Service provider2 TH).

Incentive for patient, drug observer and the heathcare facility was found an important factor to improve adherence to DOT. Incentive for patient can be provided as free transportation to visit the nearest DOT clinic. Other ways to improve DOT were creating awareness, providing proper health education to patient, counselling for patient and family, and giving patient moral support during side effects of the drugs. They also felt that there needs to be the strong political commitment to support TB program such as allocating more fund for TB program, and creating awareness through national news media.

*“DOT is main challenging part due to financial problem in transportation due to long distance, follow up, etc”* (Service provider2 TH).

#### Quality of delivery

**Fig. 2:**
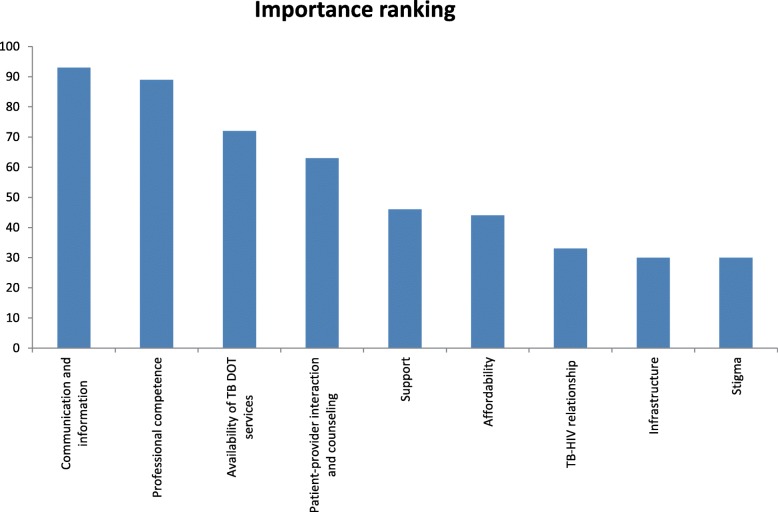
Ranking of quality dimensions for TB care by health care providers (most important to least important)

All health care providers including program officers ranked the importance of nine quality dimensions of TB care (Table 4). The result showed communication and information as the most important quality dimension with an average score of 8.3 (93%) and stigma as the least important dimension with an average score of 2.7 (30%), (Table 4 & Fig. 2).

**Table 4:** Ranking and QI score of quality dimensions (9 = most important; 1 = least important; (Note: higher the QI, need more improvement; standard QI: 0.75)

Quality dimensions	Average score	Ranking (%)	Mean score (QI)	Sub-components, QI > 0.75
Communication and information	8.33	93	0.8	Side effects of drugs, and drug storage
Professional competence	8	89	1.7	Home based treatment, physically examined, sputum examination, sputum result, close contact examination, and treatment observer
Availability of TB DOT services	6.5	72	1.5	Waiting time, same health provider, service hour inconvenient, service available, and providers available
Patient-provider interaction and counseling	5.67	63	1	Respect, sufficient time, deal with problem, discrimination, privacy and affects life
Support	4.17	46	4	Transport support and food support
Affordability	4	44	0.4	transportation cost
TB-HIV relationship	3	33	0.9	TB-HIV, prevent HIV and HIV test
Infrastructure	2.67	30	1.1	Sputum collection room, waiting room, sunlight available, provide mask, toilet usable and safe drinking water
Stigma	2.67	30	0.3	No improvement required

*DOT* Directly observed treatment, *QI* quality impact, *TB-HIV* Tuberculosis-Human immune virus

Mean of QI scores for each quality dimensions were as follows: availability of TB services (1.5), communication and information (0.8), patient-provider interaction and counseling (1.0), TB/HIV relationship (0.9), infrastructure (1.1), professional competence (1.7), affordability (0.4), support (4.0), and stigma (0.3), (Table 4).

Qualities of TB care service delivery that still need improvement from the patient’s suggestion includes reducing waiting time before being served by healthcare provider (40%), improving sputum collection room (50%), improving waiting room (40%), improving professional competence (40%), improving support for DOT provider (30%), providing transportation service (90%) and food support (70%).

#### Patient’s satisfaction

Although most patients were satisfied (94.9%)with the availability of necessary equipment, drugs, and laboratory reagents to treat TB disease, there was 6% of patient who were not happy or dissatisfied with health care provider’s ability or skill in providing TB diagnosis and treatment.

*“I think the service is excellent, I have recovered so well from the weight 55 to 65 kg, it is all because of them only, so I will give 100% only”* (TB patient5 TH).

“*DOT facility is convenient, no hospital means I won't have known the disease, we are 100% satisfied etc*”(TB patient4 TH).

## Discussion

Our study revealed that hospital-based DOT was partially implemented and this was because patients were not able to visit hospital daily basis for treatment due to long distance and financial implications. More than 90% of patient received standard regimen of anti-TB according to the guideline [1, 13]; In WHO’s definition of DOTS, the 3rd component states that “standardized treatment of 6-8 months for all confirmed sputum smear positive cases, with DOT for at least the initial 2 months”. Treatment duration was modified to 9 to 12 months for 11 TB cases (7.9%) due to perceived risk of mono/poly resistance. All patients had received TB drugs on daily basis. The dosage was prescribed in accordance with their weight range. i.e. two tablets of FDC for body weight less than 35 kg, three tablets for 35–54 kg and four tablets for more than 55 kg which is as per the NTCP guideline [1] and WHO TB treatment guideline [13].

DOT supervision should be provided for the entire course of the TB treatment if not at least for the intensive phase (2 months) as per the WHO and NTCP guideline [1, 13]. However, in this study we found hospital based DOT was provided only for 2 weeks in intensive phase. The key factors were patient unwillingness to come daily basis for treatment. According to the health worker, patients are not willing to stay in the hospital admitted as soon as they recover their health within first 2 weeks.

In the continuation phase, the hospital based DOT provided was very low (5.7%), mostly patient take medicine either with help of family member (48.9%) or by themselves (45.3%) without any supervision. It is assumed that patient would take accountability to complete the course by themselves. Study by Nirupa et al. [14] found that treatment success rates among patients treated by different DOT providers that those patients who received drugs for self administration were significantly more likely to fail to treatment or die than patients who were treated by a DOT provider [14].

On the other hand hospital based DOT depends on the patient’s type of TB disease category, which is statistically significant (*P* = < 0.05). DOT was provided more to SPPT (83.3%) and MDR-TB (100%) compared to SNPT (20%) and EPT (28.2%). Similar results were found in other countries like in Brazil [15–17], Ethiopia [18] Indonesia [19], and India [14]. A study by Lavor et al. [15], found that DOTS was partially implemented in Brazil [15]. However, in another study by Obermeyer et al. [20] had found that DOTS population coverage had a significant effect on overall treatment success rates [20].

The barriers of poor adherance to DOT was due to patients not willing to come to the clinic for DOT on daily basis due to long distance, financial crisis for traveling (cost for transportation and accomodation) and lack of support from family and friends. Similar barrier factors for DOT were reported in other studies like in Nepal [21], Brazil [17], Ethiopia [22, 23]. In addition, lack of awareness of TB and its consequences, and the belief, prompted many respondents to visit traditional healers [21]. Other barriers were patient movement from place to place, and side effects of the drug. Unlike other studies stigma [21] was not a barrier to DOT adherence in this study. Another study found that DOT can improve TB treatment adherence and protect TB patients from adverse outcomes by 25% [17]. Poor adherence by TB patients to their medication contributes not only to the worsening of their TB situation but also paves a way for the incidence of drug resistance [23].

The key enablers of DOT adherence as perceived by health workers were; by providing incentives to all TB patients, DOT provider or if the government can provide free transportation to all TB patients. In Sau Paulo, Brazil study showed the establishment of bonds between healthcare providers and patients through the introduction of incentives were some of the strength of DOT which promotes treatment adherence [24]. However, the sustainability of the incentives would be challenging, unless government keeps the strong commitment. While other studies showed that there is little evidence that DOT enhances treatment completion unless combined with other strategies. Patient-oriented community based DOT appears to be an appropriate way of addressing many of these issues [25].

Quality of TB care service delivery was found good, though few dimensions still needed improvement. Stigma was perceived as one of the least important quality dimension; this is the least ranked (30%) among nine qualities of TB care dimensions. Majority of TB patients felt that the health facility is performing well with regard to stigmatization. This may be due to either illiteracy of the people that TB is the infectious disease or due to the literacy of the people that TB can be cured if treated well. Other study result showed it might be due to the fact that patients are in a dependent situation and have a low expectation regarding this issue [26].

Most of the patient felt that TB service is useful (94.9%) for them, likewise, many of the patients were satisfied with the availability of necessary equipment, drugs and laboratory reagents to treat TB disease. The reason could be due to free service with good diagnostic and treatment facility; also there is no discrimination to TB patients. A study in India showed that although patients reported high levels of satisfaction from RNTCP DOTS services, still dissatisfaction due to financial loss in the form of loss of wages, transport charges and non-improvement of symptoms in patients were found [27]. The relapse patients and MDR-TB patients were found not satisfied in this study. Similar findings were shown in research by Getahun et al., that all persons lost to follow-up were dissatisfied [28]. The reasons for dissatisfaction are similar in many research studies like waiting time, staff’s attitudes and improvement in symptoms [27, 29, 30].

### Limitations of the study

We could not assess all 31 hospital based DOT centers in our study, due to shortages in research assistants, limited research budget and short duration of the study period. Therefore, these study findings do not represent the whole population; we recommend more research to be carried out in other hospitals too. Secondly, we could not interview other health workers of BHU-I and hospitals who managed the same TB patients during part of the treatment. The other limitations were mode of assessment by questionnaires filled out by TB patients, which is subject to recall and social desirability biases.

## Conclusion

In this study we found that the patient’s adherence to DOT was partially implemented and provider’s compliance with DOT guideline was also partially implemented in two hospitals; the coverage and the durations of DOT was also very low, therefore, there need to revise and improve DOT model and structure through patient-centered approach such as community based DOT. The key factors affecting poor adherance to DOT were; unwillingness of the patient to visit hospital daily basis due to long distance, financial implications and lack of support from family and friends during treatment. Except for quality dimension of patient stigma, there is still room for improvement needed in quality of TB care service delivery such as patient waiting time, family and friends support, though the proportion of patient satisfaction was high. Therefore, we recommend to conduct future research studies in all 31 Hospital based DOT centers for comprehensive report on DOTS strategy.

## Supplementary information

**Additional file 1.** IN-DEPTH INTERVIEW GUIDANCE FOR TB PATIENTS. The questions were developed only for this study purpose, we have adapted few questions from previous thesis and research, the total 17 open-ended questions were asked to TB patients in order to suppliment the quantitative finndings. Additionally we tried to recognize whtat were their challenges, barriers, issues and suggestions for further improvement.

**Additional file 2.** IN-DEPTH INTERVIEW GUIDANCE FOR HEALTH STAFF FOR TB-DOT. All the 16 questions were developed for this study in order to suppliment the quantitative findings; few questions were adapted from previous studies and research.

## Data Availability

The datasets used and/or analyzed during the current study are available from the corresponding author on reasonable request.

## Abbreviations

BHU: Basic Health Unit
DOT: Directly observed treatment
DOTS: Directly observed treatment, short-course
EPT: Extra pulmonary TB
JDWNRH: Jigme Dorji Wangchuk National referral hospital
KNCV: Koninklijke Nederlandse Chemische Vereniging; KNCV Tuberculosis foundation
MDR-TB: Multi-drug-resistant TB
PGH: Phuentsholing general hospital
QI: Quality impact
QUOTE: Quality of Care as seen through the Eyes of the Patient
REBH: Research Ethics Board of Health
SNPT: Smear negative pulmonary TB
SOP: Standard operating procedure
SPPT: Smear positive pulmonary TB
TB: Tuberculosis
TB/HIV: TB/ Human immuno virus
TB-CAP: TB control association program
TH: Thimphu
WHO: World health organization
XDR-TB: Extensively drug-resistant TB

## References

[1] Bhutan N (2016). National guidelines for the management of tuberculosis.

[2] WHO. WHO _ Tuberculosis_factsheets 2017. 2017.

[3] Health Bulletin, M. Ministry of Health, Bhutan. (MoH Bhutan, 2017). http://www.moh.gov.bt/annual-health-bulletin/.

[4] World Health Organization (2017). Global Tuberculosis Report 2017-main text.

[5] WHO. WHO country profile. 2–3. 2017.

[6] Darnal JB, Swaddiwudhipong W. Multidrug-resistant Tuberculosis Patients in Bhutan, August 2011 to July 2012; 2013. p. 6–10.

[7] Dusenbury L, Brannigan R, Falco M, Hansen WB (2003). A review of research on fidelity of implementation: implications for drug abuse prevention in school settings. Health Educ Res.

[8] Carroll C (2007). A conceptual framework for implementation fidelity. Implement Sci.

[9] Training, W. H. O. on behalf of the S. P. for R. and & in Tropical Diseases (2014). Implementation research toolkit.

[10] Peters DH, Adam T, Alonge O, Agyepong IA, Tran N (2014). Republished research: implementation research: what it is and how to do it. Br J Sports Med.

[11] Brownson RC, Colditz GA, Proctor EK (2012). Dissemination and implementation research in health: translating science to practice.

[12] Massaut S, ven den Broek J, ven der Kwaak A (2007). QUOTE TB LIGHT.

[13] World Health Organization (2010). Treatment of tuberculosis: guidelines.

[14] Nirupa C (2005). Evaluation of directly observed treatment providers in the revised National Tuberculosis Control Programme. Indian J Tuberc.

[15] Lavôr DC, Pinheiro JS, Gonçalves MJ (2016). Evaluation of the implementation of the directly observed treatment strategy for tuberculosis in a large city. Rev da Esc Enferm.

[16] Portela MC (2014). Tuberculosis control program and patient satisfaction, Rio de Janeiro, Brazil. Rev Saude Publica.

[17] Reis-Santos B (2015). Directly observed therapy of tuberculosis in Brazil: associated determinants and impact on treatment outcome. Int J Tuberc Lung Dis.

[18] Kim SS (2015). Assessing implementation fidelity of a community-based infant and young child feeding intervention in Ethiopia identifies delivery challenges that limit reach to communities: a mixed-method process evaluation study global health. BMC Public Health.

[19] Probandari A, Utarini A, Hurtig AK (2008). Achieving quality in the directly observed treatment short-course (DOTS) strategy implementation process: a challenge for hospital public-private mix in Indonesia. Glob Health Action.

[20] Obermeyer Z, Abbott-Klafter J, Murray CJL (2008). Has the DOTS strategy improved case finding or treatment success? An empirical assessment. PLoS One.

[21] Marahatta SB (2020). Barriers in the access, diagnosis and treatment completion for tuberculosis patients in central and western Nepal: a qualitative study among patients, community members and health care workers. PLoS One.

[22] Fiseha D, Demissie M. Assessment of Directly Observed Therapy ( DOT ) following tuberculosis regimen change in Addis Ababa , Ethiopia : a qualitative study. BMC Infect Dis. 2015:1–9. 10.1186/s12879-015-1142-2. 10.1186/s12879-015-1142-2 PMC4590704 26423277

[23] Gugssa Boru C, Shimels T, Bilal AI. Factors contributing to non-adherence with treatment among TB patients in Sodo Woreda, Gurage zone, Southern Ethiopia: A qualitative study. J Infect Public Health. 2017. 10.1016/j.jiph.2016.11.018. 10.1016/j.jiph.2016.11.018 28189508

[24] de Queiroz EM, De-La-Torre-Ugarte-Guanilo MC, Ferreira KR, Bertolozzi MR (2012). Tuberculosis: limitations and strengths of directly observed treatment short-course. Rev Lat Am Enfermagem.

[25] Thomas C (2002). Best dissertation section. J Manag Med.

[26] Sugiharto J (2012). Assessing the perceived quality of care.

[27] Shalini Srivastav HM (2014). Satisfaction levels among patients availing DOTS services in Bundelkhand region (UP), India: Evidence from patient exit-interviews.

[28] Getahun B, Nkosi ZZ (2017). Satisfaction of patients with directly observed treatment strategy in Addis Ababa, Ethiopia: a mixed-methods study. PLoS One.

[29] Palha PF (2012). Access to healthcare services for tuberculosis: analysis of patient satisfaction. Rev Esc Enferm USP.

[30] Gupta S (2015). Evaluation of patient satisfaction level undergoing dots therapy in meerut District of Uttar Pradesh. J Adv Med Dent Sci Res.

