# Supplementary material for: Implementation fidelity of hospital based directly observed therapy for tuberculosis treatment in Bhutan: mixed-method study
Source: BMC Public Health. 2020 Apr 19;20:533. doi: 10.1186/s12889-020-08666-w (PMC7168849; doi:10.1186/s12889-020-08666-w)
Supplement: Supplementary file 1 — Additional file 1. IN-DEPTH INTERVIEW GUIDANCE FOR TB PATIENTS. The questions were developed only for this study purpose, we have adapted few questions from previous thesis and research, the total 17 open-ended questions were asked to TB patients in order to suppliment the quantitative finndings. Additionally we tried to recognize whtat were their challenges, barriers, issues and suggestions for further improvement. [file 12889_2020_8666_MOESM1_ESM.pdf]

Additional file 1: IN-DEPTH INTERVIEW GUIDANCE FOR TB PATIENTS

Date of interview:
Place of interview:
Time of interview:
Interviewer:
Code of informant:

Introduction

Before we start, let me introduce myself; my name is Kunzang Dorji, you can call me Kunzang. I am currently pursuing master degree in Public Health at Universitas Gadjah Mada. I am doing my research on TB in Phuntsholing Hospital with the title ”Assessing Implementation Fidelity of TB-DOT provider in JDWNR-Hospital and PGH in Bhutan”. The result from this study is expected to find barriers of TB-DOT in this Hospital and to propose some solutions to it. Therefore, the information generated will be used to plan better TB treatment services for the patients.

I want you to know that your participation is completely voluntary. You do not have to answer questions if you don’t want to – it will not influence the treatment you receive currently. I understand that you may not know the answers to all questions but please answer honestly to the best of your knowledge. The information will be treated confidential and available only to the study staff. You should also know that I am not a doctor and I will not be able to provide any medical care.Are you willing to be respondent in this study?

To avoid errors and or incomplete information in data analyzing, can we record this interview? This recording will not be given to someone else outside the research team. Clear information such as name, address, age and other detailed information will only be known by researchers.

I would like to appreciate your help in responding to this interview. If you are comfortable let us start the discussion with:

1. In your opinion, how well do health workers communicate with you during the treatment of your disease? (Probes: is it harsh, polite, etc).
2. Do you know you’re treated for TB or for MDR-TB, which one?
3. Do you know how long is your treatment duration will last?
4. Do you take your medication while you are being directly observed? Is treatment convenient for you in terms of the time and location?

5. Do you take your medication regularly? How many doses were missed during the past month?
6. What are the main issues that affect when you follow the treatment plan? (For example, did you have any adverse effects; have there been problems with deliveries of medicines or DOT services, transport or problems at work?)
7. Do you know about the schedule for follow-up sputum examinations and the final review?
8. Have you ever been treated for TB in the past? Can you tell me how you became infected with TB?
9. Have you received health information about your disease, treatment and prognosis? Have you understood the information that they have given to you?
10. How do you think the overall structure, comfortableness of the health care facility where you diagnosed as you have TB and started treatment?
11. Since you have been on treatment for TB, what problems or barriers do you encounter while seeking treatment under this arrangement? Probes: (problems in terms of language barrier for communication, or financial, was there lack of moral support, lack of nutrition, etc.)
12. Do you feel that the health-care services satisfactorily address the emotional and spiritual suffering that results from the disease and its treatment?
13. Do you feel that your privacy is respected when you interact with the health services?
14. What do your families and people in your communities think about TB? Do you feel stigmatized or discriminated against because of your disease?
15. At what extent you satisfied with the service provided to you while you were on following up your treatment? (Probe: How many percent?)
16. What do you suggest to improve TB patients' satisfactions with service?
17. Is there anything more you would like to add?
