# Supplementary material for: Implementation fidelity of hospital based directly observed therapy for tuberculosis treatment in Bhutan: mixed-method study
Source: BMC Public Health. 2020 Apr 19;20:533. doi: 10.1186/s12889-020-08666-w (PMC7168849; doi:10.1186/s12889-020-08666-w)
Supplement: Supplementary file 2 — Additional file 2. IN-DEPTH INTERVIEW GUIDANCE FOR HEALTH STAFF FOR TB-DOT. All the 16 questions were developed for this study in order to suppliment the quantitative findings; few questions were adapted from previous studies and research. [file 12889_2020_8666_MOESM2_ESM.pdf]

Additional file 2: IN-DEPTH INTERVIEW GUIDANCE FOR HEALTH STAFF FOR TB-DOT

Date of interview:
Place of interview:
Time of interview:
Interviewer:
Name of informant:
Position:

Introduction

Before we start, let me introduce myself; my name is Kunzang Dorji, you can call me Kunzang. I am currently pursuing master degree in Public Health at Universitas Gadjah Mada. I am doing my research on TB in Phuntsholing Hospital with the title "Assessing Implementation Fidelity of TB-DOT provider in PGH in Bhutan". The result from this study is expected to assess gaps and barriers of TB-DOT in Hospitals and to propose some solutions to it. Are you willing to be respondent in this study?

To avoid errors and or incomplete information in data analyzing, can we record this interview? This recording will not be given to someone else outside the research team. Clear information such as name, address, age and other detailed information will only be known by researchers.

I would like to appreciate your help in responding to this interview. If you are comfortable let us start the discussion with:

1. According to you, how do you feel about the TB caseload in this facility?
2. Can you briefly describe how TB treatment is being provided in this hospital for TB patients?
3. What are the problems to DOTS strategy?
4. How TB patients select their treatment supporter?
5. How do you know either TB patients are being satisfied or not? Is there anything to monitor the patient's satisfaction towards the service that you provide to them?
6. In your opinion, what are the factors that may contribute to poor adherence towards TB treatment in intensive and continuation phase?

7. What do you suggest to improve TB patients' adherence towards DOT?
8. What are the comprehensiveness policy available ? (Probe: National health policy, NTCP guideline, Hospital guideline on DOT, etc.)
9. Your opinion on implementation of DOT in this hospital (Probe: do you think is running well or Not, why? What could be the resaons if any?)
10. Do you strictly follow the DOT SOP to treat TB patient or do you treat base on expereince? Is the DOT procedure easy to keep in mind, how do you do?
11. What are the strategies of facilating available from the NTCP or government to your Hospital in implementating the DOT? (Training, logistic supplies, incentives if any, etc.)
12. Do you feel the difficulty of patient while taking drugs, how do you support them morally to take medicine?
13. According to you how difficult is to treat patient under DOT?
14. What are the problems, barriers and chalenges do you face to provide DOT?
15. According to you, what would be the solution of those needs to be improved?
16. Is there anything more you would like to add?

Thank you so much for the willingness to participate in this interview, I apologize if there were something unpleasant to you. If there are other necessary information, are you willing to be invited again? Thank you.
